# The Forgotten Allotrope: γ-Sulfur Stabilization in Carbon Matrices for Energy Storage Applications

**DOI:** 10.3390/ma19163537

**Published:** 2026-08-20

**Authors:** Marlena Bytniewska, Dimitrios A. Giannakoudakis, Mariusz Barczak

**Affiliations:** Institute of Chemical Sciences, Faculty of Chemistry, Maria Curie-Sklodowska University, 20-031 Lublin, Poland; marlena.bytniewska@mail.umcs.pl (M.B.); dimitrios.giannakoudakis@mail.umcs.pl (D.A.G.)

**Keywords:** γ-sulfur, gamma sulfur, lithium–sulfur batteries, polysulfides, porous carbon, nanoconfinement, XRD

## Abstract

**Highlights:**

- Lithium-ion batteries are approaching performance limits, indicating the need for new successor technologies.
- Sulfur emerges as a promising cathode material for metal–sulfur batteries.
- Among sulfur allotropes, γ-sulfur is particularly attractive, but challenging to stabilize.
- Pore geometry and carbon surface chemistry govern γ-sulfur stabilization in carbon matrices.

**Abstract:**

Lithium–sulfur (Li-S) batteries are widely regarded as one of the most promising candidates for next-generation electrochemical energy storage, owing to their very high theoretical energy density and reliance on abundant, low-cost elements. However, the practical deployment of Li-S technology remains severely constrained by the polysulfide shuttle effect, originating from the dissolution, migration and parasitic redox cycling of lithium polysulfide intermediates, which leads to rapid capacity fading, low coulombic efficiency and incompatibility with industrial carbonate-based electrolytes. Recent reports on the formation and stabilization of γ-sulfur, a rare monoclinic allotrope, within porous carbon matrices have identified a prospective direction in sulfur electrochemistry, theoretically enabling polysulfide-free cycling and improved stability, also in conventional carbonate electrolytes. These findings challenge the long-held assumption that polysulfide formation is unavoidable in sulfur cathodes and suggest that control over sulfur allotropy and nanoconfinement, as well as carbon–sulfur chemistry, may unlock previously inaccessible performance and integration windows for metal–sulfur batteries, including most technologically advanced Li-S batteries. Based on recent studies, this Perspective article critically evaluates the evidence for γ-sulfur stabilization in carbon hosts, discusses the interplay between pore geometry, carbon surface chemistry and sulfur speciation, and finally identifies key knowledge gaps.

## 1. Introduction

Over the last decade, annual demand for Li-ion batteries has already reached the level of 700 GWh and is projected to climb toward 4.7 TWh by 2030, driven predominantly by electromobility market needs [1]. On the grid side, installed electrochemical storage is expected to expand from tens of gigawatts today to several hundred gigawatts by 2030, with some net-zero scenarios requiring close to 1 TW of grid-scale battery capacity within this decade [2]. This rapid electrification of transport and the large-scale integration of intermittent renewable energy sources have created an unprecedented demand for high-energy-density electrochemical storage systems to power electric vehicles, portable electronics, and grid-scale infrastructure, as shown in Table 1. Lithium-ion batteries (Li-ion) currently dominate this landscape due to their favorable combination of specific energy, power capability, and cycle life, as well as a well-developed manufacturing and supply chain ecosystem [1,2,3,4,5]. It is estimated that Li-ion battery demand will be growing ~25% annually to reach around 4700 GWh by 2030.

Li-ion emerged in the late 20th century, resulting from the pioneering work on intercalation electrodes and non-aqueous electrolytes [6,7,8] and was first commercialized by Sony in 1991 for portable electronics [9]. Since then, continuous advances in layered transition-metal oxide cathodes, graphite and silicon-based anodes, and electrolyte formulations have enabled substantial gains in energy density, power capability and cycle life, establishing Li-ion as the dominant rechargeable battery technology for consumer devices and electric vehicles [9,10,11]. However, Li-ion-based technologies, while commercially mature, are approaching theoretical performance limits: graphite anodes already operate near their theoretical capacity of 372 mAh g^−1^, and nickel–manganese–cobalt (NMC)-layered oxide cells reach ~300 Wh kg^−1^ [12,13].

In contrast to conventional intercalation-type cathodes (based on topotactic ion insertion into a host lattice) employed in Li-ion, electrochemical energy storage proceeding via sulfur-sulfur bond breaking and forming started to be developed from the 1990s [14,15,16]. Sulfur emerged there as an advantageous cathode material and was coupled with various metal anodes to form a broader class of metal–sulfur systems. In these cells, the overall cell reaction involves the conversion of elemental sulfur into the corresponding metal sulfides (M_x_S_y_) via multi-electron redox processes mediated by the formation of soluble polysulfide intermediates. This underpins exceptionally high theoretical capacities and the exploitation of earth-abundant, low-cost active materials [15,17,18,19]. Representative realizations of this concept resulted in the development of lithium–sulfur (Li-S), magnesium–sulfur (Mg-S), potassium–sulfur (K-S), and aluminum–sulfur (Al-S) batteries, which are all built of the sulfur-based cathode, yet employ markedly different anode chemistries, electrolyte environments, ion-transport characteristics and reaction mechanisms, resulting in distinct electrochemical performance, stability regimes and target application domains [19].

Among various sulfur-based batteries, Li-S batteries are most intensively investigated, attracting remarkable attention as possible Li-ion technological successors for high-energy applications [15,18]. The complete electrochemical reduction of elemental sulfur to lithium sulfide (S → Li_2_S) involves a two-electron transfer, yielding a theoretical specific capacity of 1675 mAh g^−1^ and an energy density of ~2600 Wh kg^−1^ [20], roughly five times that of conventional Li-ion systems (vide supra). In contrast to cathode materials in conventional lithium-ion batteries, which rely on scarce and geopolitically sensitive elements such as cobalt and nickel, sulfur is abundant, non-toxic, and cheap, making Li-S batteries more attractive from a circular-economy and cost-reduction perspective [18]. This trend is even more pronounced for aluminum–sulfur batteries, where both electrode components are widely available and inexpensive. A comparative overview of the key performance and design of Li-ion, Li-S and Al-S batteries is presented in Table 2.

As can be seen in Table 2, unlike the rapid commercial trajectory of Li-ion batteries (started from the 1990s), Li-S technology has so far failed to translate laboratory promise into practical deployment. This is primarily due to intrinsic limitations of elemental sulfur: extremely low electronic conductivity and tendency to form soluble lithium polysulfides, which migrate and redeposit in the Li-S cell, causing continuous loss of active material, self-discharge, lowering of coulombic efficiency, and rapid capacity fading (so-called shuttle effect [14,15]. These issues are discussed in detail in Section 2.

Both above-mentioned challenges (low electronic conductivity and polysulfide formation) can be addressed through two conceptually distinct strategies, which may also be potentially implemented simultaneously within a single cathode design. These strategies are mainly based on controlled sulfur allocation within a conductive host by appropriate structural and chemical confinement. This may ensure efficient electron transport and a more homogeneous redox reaction throughout the electrode as well as mitigate the formation and migration of soluble polysulfides.

Recent results reported over the last few years indicate that certain sulfur species (short-chained sulfur) and allotropes (particularly γ-sulfur) are particularly well suited for being used as cathode materials in metal–sulfur batteries. Therefore, Section 3 briefly summarizes the characteristics of the most common sulfur allotropes, including γ-sulfur. Specific synthetic protocols for such conductive sulfur–carbon composites that lead to the in situ formation of γ-sulfur are discussed in Section 4, together with strategies for stabilizing γ-sulfur within carbon matrices. Finally, Section 5 presents materials chemistry-driven perspectives and conclusions, followed by a concise list of recent literature to facilitate deeper exploration of these topics by the reader.

## 2. The Promises and Bottlenecks of Li-S Batteries

Lithium–sulfur batteries (Li-S) occupy a unique position within the metal–sulfur family due to the very high sulfur theoretical capacity and a theoretical cell-level energy density (1675 mAh g^−1^ and ~2600 Wh kg^−1^, respectively; cf. Table 2) far exceeding typical values reported for state-of-the-art Li-ion batteries. Such high metrics stem from the nature of the S_8_ → Li_2_S redox process, involving a two-electron transfer transforming elemental sulfur into lithium sulfide in contrast to the intercalation chemistry of layered transition-metal oxides. Despite these high metrics, Li-S batteries still remain at a pre-commercial stage, and their deployment is hindered by a set of intertwined material-related and electrochemistry-related bottlenecks. The most challenging ones are related to physicochemical properties of sulfur and its discharge products, in particular the formation of soluble polysulfides (the shuttle effect), large volume changes, and the very low electronic and ionic conductivity of sulfur-containing phases [14,15,26,30].

**Figure 1:**
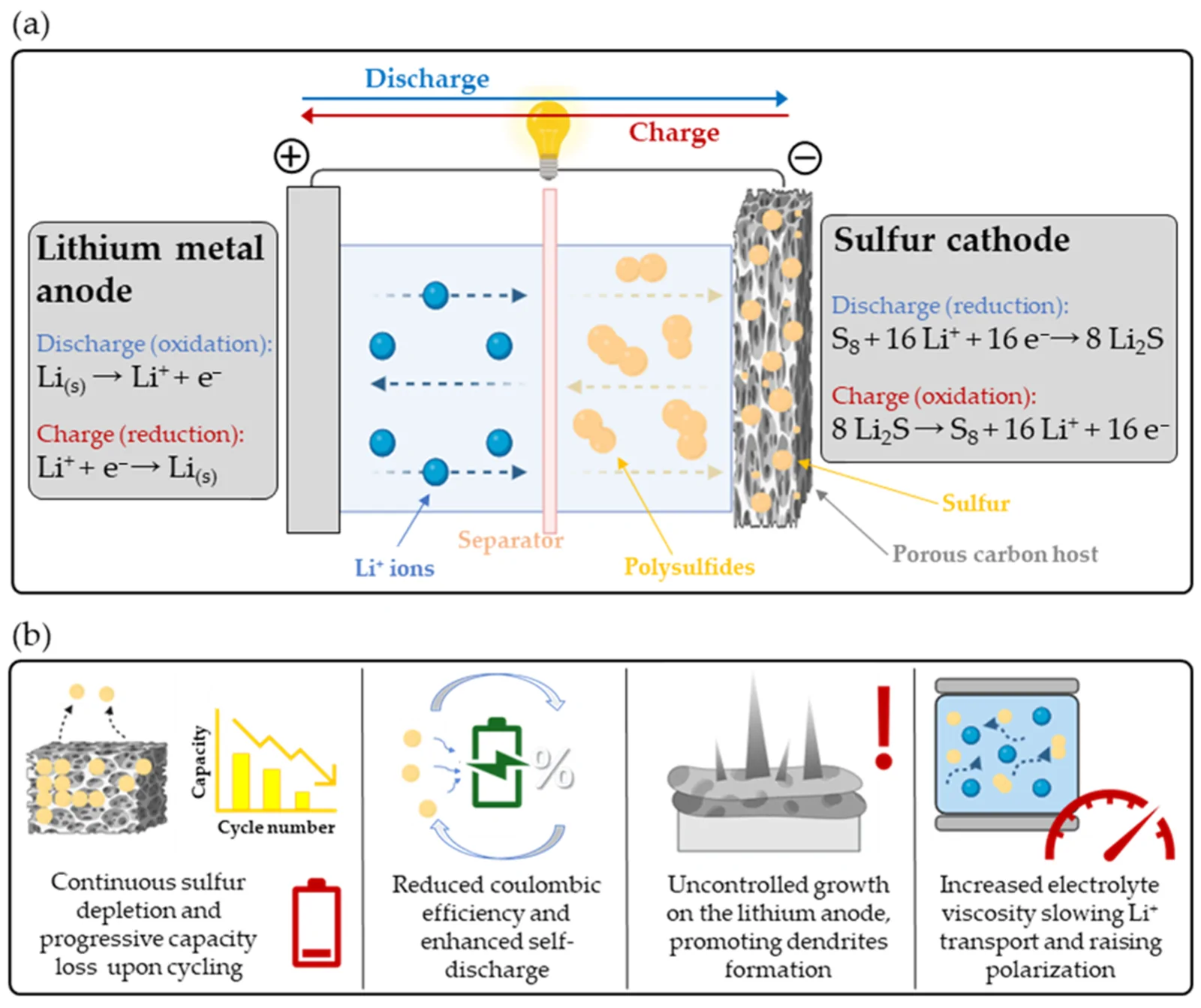
Schematic illustration of polysulfide shuffling effect (**a**) and major drawbacks associated with the polysulfide shuttle in Li-S batteries (**b**).

During discharge, elemental sulfur (e.g., in the form of S_8_ rings) is sequentially reduced in two stages. In the first stage, sulfur is converted into soluble long-chain lithium polysulfides (Li_2_S_8_, Li_2_S_6_, Li_2_S_4_), typically at potentials around 2.3–2.4 V vs. Li/Li^+^. In the second stage, these intermediates are further reduced to insoluble final discharge products Li_2_S_2_ and Li_2_S at about 2.0–2.1 V vs. Li/Li^+^. From an electrochemical perspective, soluble polysulfides act as redox mediators that enable deep sulfur utilization and high capacity, but a direct consequence of this multi-step redox chemistry and the presence of soluble intermediates is that the metal–sulfur cells become prone to pronounced migration of polysulfides and undesirable side reactions at the lithium anode, known as polysulfide shuttling. The polysulfide shuttle effect can be briefly described as the cyclic generation, dissolution, migration and re-deposition of soluble lithium polysulfides between the sulfur cathode and the lithium anode, accompanied by side reactions at the anode and secondary redox processes, which together cause active-material loss, self-discharge and electrolyte degradation [14,26,31]. Polysulfides produced at the cathode are dissolved and driven by a concentration gradient and the internal electric field, diffuse through the separator toward the lithium anode, where they undergo chemical reduction to short-chain Li_2_S_2_/Li_2_S sulfides, thickening and roughening of the solid-electrolyte interphase (SEI). Of course, upon charging, part of these species migrates back to the cathode, but instead of being quantitatively oxidized to S_8_, they participate in multiple side reactions with remaining polysulfides and the electrolyte, sustaining the shuttle cycle and accelerating performance decay. This cyclic polysulfide transport leads to several detrimental effects, schematically presented in Figure 1:

- Continuous depletion of sulfur from the cathode region and progressive capacity loss upon cycling [14,15];
- Reduced coulombic efficiency and enhanced self-discharge [14,31];
- Uncontrolled growth and chemical modification of the solid-electrolyte interface (SEI) on the lithium anode, and on dendrite formation [32,33];
- Higher electrolyte viscosity induced by the presence of polysulfides, which slows Li^+^ transport and increases polarization [14,30,34,35].

From the electrolyte point of view, there is an additional and often underappreciated barrier. The ether-based electrolytes predominantly used in Li-S research (e.g., 1,3-dioxolane or dimethoxyethane) tolerate the polysulfide intermediates reasonably well; however, ether-based electrolytes are highly volatile and flammable. Despite being predominantly “academic” electrolyte choices, they are incompatible with commercial lithium-ion manufacturing lines [14,15,36,37,38]. In contrast, carbonate-based electrolytes, which are the industrial standard for Li-ion batteries (e.g., ethylene carbonate, dimethyl carbonate or ethyl methyl carbonate), are fundamentally incompatible with conventional Li-S cathodes, because polysulfide species irreversibly react with electrophilic carbonate solvents, leading to ring-opening decomposition products and rapid cell failure [37,39,40,41]. This incompatibility is one of the most fundamental barriers to sulfur cathodes [37,39].

Beyond the shuttle effect, the next bottleneck of Li-S batteries is the microscopic nature of charge carriers in sulfur and its discharge products. Both S_8_ and Li_2_S are essentially electronic and ionic insulators; with room-temperature sulfur exhibiting an electronic conductivity of about 10^−30^ S cm^−1^, whereas Li_2_S shows a higher but still very low conductivity of approximately 10^−13^ S cm^−1^ [14,15,42,43]. Another critical aspect is significant volume change accompanying the S_8_ (d ≈ 2.0 g cm^−3^) → Li_2_S (d ≈ 1.7 g cm^−3^) conversion, thus generating substantial mechanical stress within the cathode layer upon cycling, which results in cracking, fracture of agglomerates, loss of contact with the current collector, and detachment of active material [14,36,44].

As a consequence, sulfur itself cannot be used as cathode unless it is incorporated within a conductive host, which provides continuous electron pathways and short ionic diffusion lengths. Incorporating sulfur into nanoporous carbon hosts (e.g., activated carbons, carbon nanotubes, graphene-based materials) provides a continuous conductive backbone and short diffusion pathways, improving reaction kinetics. At the same time, appropriately designed host may act as confinement media for soluble lithium polysulfides, suppressing their diffusion into the bulk electrolyte and the associated shuttle effect that causes active-material loss, self-discharge, and undesirable reactions at the Li anode [14,15,45,46]. Hence, rational design of sulfur–carbon composite cathodes-with controlled porosity, tailored surface chemistry and optimized sulfur loading is a prerequisite for any practical implementation of metal–sulfur batteries and remains one of the critical research directions of material chemists working in this area.

## 3. γ-Sulfur: A Forgotten Allotrope at the Center of Li-S Battery Research

**Figure 2:**
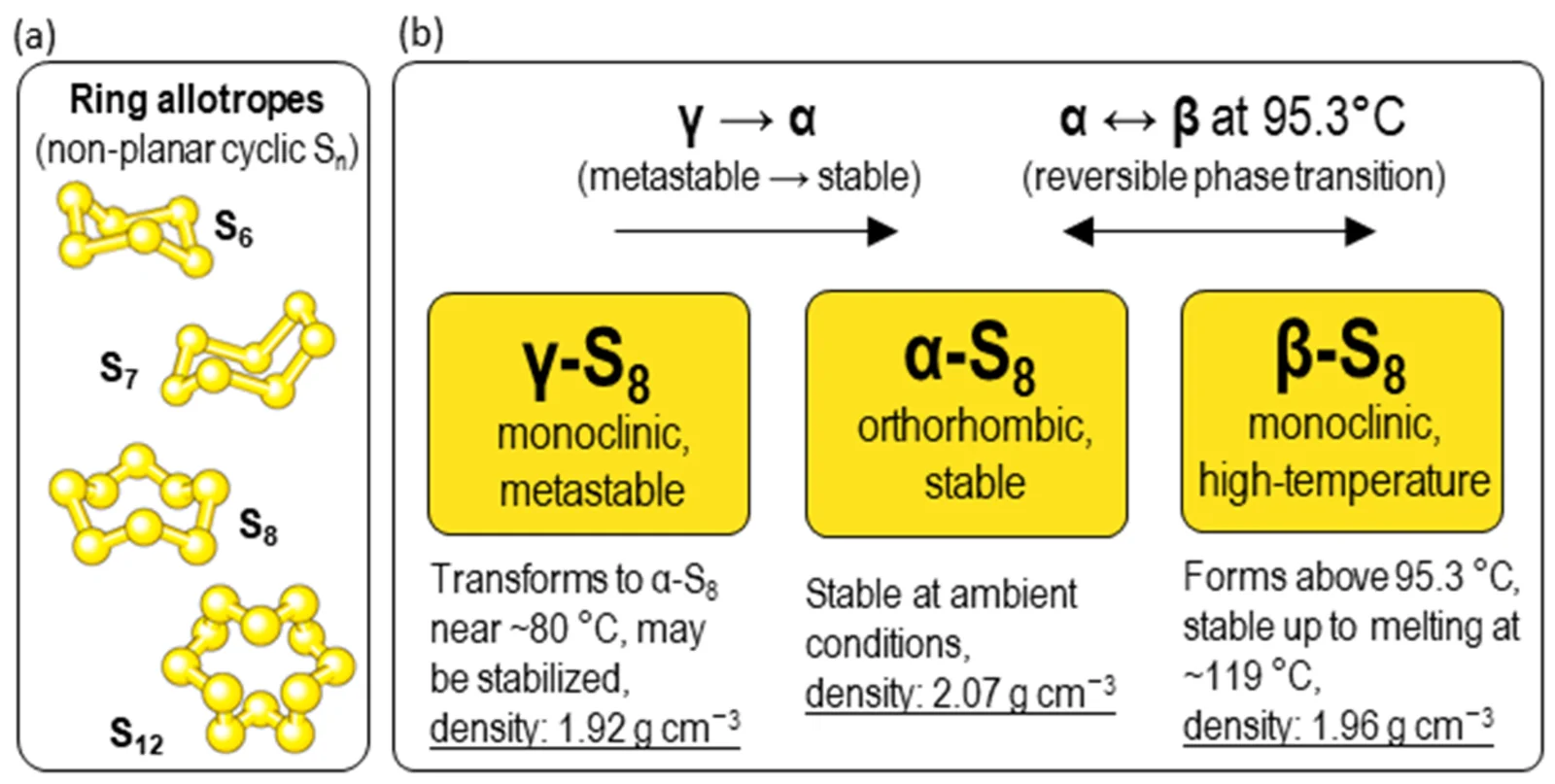
Examples of non-planar cyclic sulfur ring allotropes: S_6_, S_7_, S_8_, and S_12_ (**a**); temperature-dependent relationships between the α-, β-, and γ- polymorphs of cyclo-S_8_ sulfur (**b**).

Sulfur is one of the most structurally diverse elements in the periodic table, exhibiting over thirty known allotropes; many of these allotropes occur in multiple polymorphs, i.e., different crystal structures built from the same covalently bonded S_n_ molecules) [47,48,49]. Under ambient conditions, elemental sulfur does not exist as isolated atoms, but predominantly as cyclic crown-shaped rings of different sizes (S_6_–S_18_) or chained “catena” structures, yet S_8_ remains the dominant motif (either ringed or cyclic) at room temperature [47,48,50]. This thermodynamically stable S_8_ form may exist in three forms/polymorphs that differ only in the way the rings are packed in the crystal: orthorhombic α-sulfur, monoclinic β-sulfur, and γ-sulfur [49]. Orthorhombic α-S_8_ (with a density of 2.07 g cm^−3^) at 95.3 °C α-S_8_ transforms reversibly into monoclinic β-S_8_, which is stable above 95.3 °C to ~119 °C (melting point). Both α- and β-forms share the same S_8_ ring molecule; they differ exclusively in the packing arrangement of these rings within the crystal lattice (cf. Figure 2). The third cyclic S_8_ allotrope, γ-sulfur, is also monoclinic, but adopts a distinct space group with a different ring-packing geometry compared to β-S_8_. At ambient temperature, γ-S_8_ is metastable and is spontaneously converted to the α-S_8_ form [47,49]. Interestingly, the γ-sulfur occurs naturally in the rare mineral, rosickyite, which is sufficiently stable to persist under natural geological conditions, despite being metastable relative to the thermodynamically stable α-sulfur.

For a long time, γ-sulfur’s instability and scarcity made it seem like nothing more than a crystallographic curiosity. It was not expected that this rare allotrope would one day emerge as a highly desirable phase for lithium–sulfur batteries. This occurred in 2013, when Moon et al. [51] reported that, instead of the usual orthorhombic phase, monoclinic S_8_ could form after heat treating sulfur inside a confined environment, specifically, within carbon nanotubes deposited on a porous alumina surface. The authors suggested that the proposed synthesis protocol favored the formation of the monoclinic phase. Additionally, the confined nanopore structure appeared to prevent the usual transition from monoclinic to orthorhombic sulfur, allowing the monoclinic form to remain structurally stable.

Importantly, Moon et al. showed that the pore-confined monoclinic sulfur phase was not merely a scientific curiosity and had a real impact on the performance of Li-S batteries, which exhibited single-plateau discharge curves and reached reversible capacities close (~1520 mAh g^−1^, 91%) to the theoretical limit for sulfur (~1675 mAh g^−1^). Remarkably, the obtained electrode material maintained stable operation for up to 1000 cycles, retaining 75.8% of the initial capacity (1138 mAh g^−1^ at a discharge rate of 5 C and a charge rate of 2 C). These findings were some of the first to prove that a monoclinic S_8_ phase can be stabilized at room temperature inside a solid host and can operate reliably in real battery systems. The authors demonstrated the presence of monoclinic sulfur in selected regions of the composite, identified using ex situ XRD and HRTEM/FFT analysis. However, no solid mechanistic explanation was proposed on how this particular sulfur allotrope could contribute to the remarkable electrochemical performance observed. Therefore, the single-plateau behavior observed by Moon et al. and later γ-S studies should not be taken as definitive evidence that the polysulfide shuttle was completely eliminated. Similar one-plateau behavior and long-life cycling were also reported for Li–S cells, where polysulfide formation is strongly mitigated without reporting the presence of γ-sulfur allotrope. In fact, the clear mechanistic evidence linking γ-sulfur directly to a direct S → Li_2_S pathway is still missing. Therefore, the single-plateau behavior, while being a strong indicator of a highly constrained, shuttle-poor reaction environment that is compatible with γ-S phase, cannot yet be considered an intrinsic or exclusive property of the presence of monoclinic sulfur.

Moreover, the work of Moon et al. [51] together with earlier studies by Ji et al. [46] and Xin et al. [52] challenged the long-standing assumption that the formation of polysulfides in lithium–sulfur batteries is inevitable. From a materials chemistry and engineering perspective, it also renewed interest in γ-sulfur-based cathodes, while leaving open the question of whether the unusual electrochemical behavior is specific to the monoclinic phase itself or to the nanoconfinement effect—a topic explored in more detail in Section 4.

## 4. γ-Sulfur Stabilization in Carbon Matrices: State-of-the-Art

As discussed above, control over sulfur allotropy (in particular, confinement of gamma sulfur within porous carbon matrices) has emerged as one of the strategies assumed to shift the polysulfide-mediated conversion towards more direct S → Li_2_S conversion, thus opening new pathways to improved stability and electrolyte compatibility. Xin et al. [52] designed a conductive microporous carbon matrix able to trap small, metastable sulfur molecules (S_2_–S_4_) within ultra-small micropores, thereby blocking the formation and migration of longer-chain polysulfides. This electrode material was obtained by coating multi-walled carbon nanotubes with a microporous carbon layer. Sulfur was inserted into the micropores by melt diffusion, and its successful incorporation was confirmed by nitrogen sorption, which revealed almost a complete filling of micropores (micropore pore volume dropped from 0.46 to 0.04 cm^3^ g^−1^). HRTEM and pore size analysis indicated that only chain-like S_2_–S_4_ molecules could fit within these tiny pores, while larger S_8_ rings were too bulky to enter. HRTEM did not evidence crystalline sulfur in the microporous carbon, while Raman spectra also lacked the typical cyclo-S_8_ peaks, further hinting at altered sulfur speciation, though distinguishing between S_2_, S_3_, and S_4_ was based mostly on modeling, not direct measurement. This work was one of the first to clearly connect sulfur molecule size with pore size, yet not allotrope speciation. It was achieved by combining several complementary ex situ techniques (HRTEM, SEM, Raman, and nitrogen sorption).

The fabricated electrodes behave differently from a classic sulfur–carbon black composite. In an ether-based electrolyte, the latter displayed two classical discharge plateaus and low initial efficiency due to polysulfide loss. In contrast, the former exhibited just a single discharge plateau at ~1.9 V, with a high initial capacity (~1667 mAh g^−1^) and a single charge plateau at ~2.0 V, consistent with a direct S_2_–S_4_ → Li_2_S conversion, skipping the usual S_8_ → Li_2_S_4_ step. Most importantly, it was demonstrated that the fabricated cathode maintained a single plateau and high capacity over 200 cycles in carbonate electrolyte, while the classic electrode quickly degraded. Raman spectroscopy of discharged electrodes shows Li_2_S features only inside the carbon matrix, indicating both sulfur and Li_2_S remain strictly confined to the micropores. Scanning electron microscopy analysis of the lithium anodes supported the claim that trapping small S_2_–S_4_ molecules in micropores nearly eliminates the polysulfide shuttling [52]. However, as mentioned before (vide supra), the mechanistic evidence for this voltage profile lacks a clear explanation of the role of nanoconfined sulfur.

Li et al. [53] investigated the idea of trapping small sulfur chains within microporous carbons a step further by deliberately separating the roles of confined S_2_–S_4_ and surface S_8_ in an ordered microporous carbon host (S_BET_ = 876 m^2^ g^−1^ and V_T_ = 0.93 cm^3^ g^−1^, cf. Table S1 in Supplementary Materials), loading sulfur predominantly into micropores < 0.5 nm via melt-diffusion. Two types of sulfur were then observed: chain-like S_2_–S_4_ species in the micropores, and ring-like S_8_ species mainly at or near the surface. After washing, only the confined S_2_–S_4_ fraction remained. Electrochemical tests showed a single discharge plateau at around 1.8 V and capacities exceeding 1000 mAh g^−1^ in both ether and carbonate electrolytes. This stability was explained by the fact that the micropores accommodate short sulfur chains but exclude solvent molecules (which was supported by DFT modeling), thereby blocking the formation and dissolution of long-chain polysulfides and protecting carbonate solvents from unwanted side reactions. However, when both S_2_–S_4_ and S_8_ were present in a reference carbon sample, a typical polysulfide behavior and worse performance in carbonate electrolyte was observed. This demonstrated that even small amounts of S_8_ species outside the micropores can dominate the overall electrochemistry behavior. Notably, this work did not address the allotropy of sulfur directly; instead, it explained the results purely in terms of confinement-driven suppression of polysulfides, which was conveniently supported by DFT calculations.

It was Moon et al. [51] who showed one of the first clear demonstrations that monoclinic sulfur can be stabilized at room temperature by carefully engineering its nano-environment. In the proposed approach, anodic aluminum oxide membranes with straight, well-aligned nanochannels were used as a template (cf. Table S1, Supplementary Materials). Then, the inner surfaces of these pores were coated with a thin ~3 nm thick layer of carbon nanotube shell. Sulfur was then drawn into these shells via capillary forces at 155 °C, followed by a key heat treatment at 400 °C. Finally, a thin platinum layer is sputtered onto one side to seal the pores, thereby preventing sulfur loss and maintaining the structure’s mechanical stability. It was shown that orthorhombic S_8_ forms in the core of the sulfur nanowires, while sulfur close to the carbon walls takes on a monoclinic phase that surprisingly stays stable at room temperature. This is notable since monoclinic S_8_ is typically stable only between about 95 °C and 119 °C [47], reverting to the orthorhombic form upon cooling. The persistence of monoclinic domains suggests that the carbon walls strongly influence the crystal structure, likely by templating and kinetically trapping the monoclinic phase. However, the study did not specify the relative shares of monoclinic versus orthorhombic sulfur. It was also shown that the γ-sulfur phase is directly tied to excellent Li-S battery cycling performance. Electrochemically, the cells showed single discharge plateaus at ~1.97 V with specific capacities up to ~1520 mAh g^−1^ at 0.5 C (i.e., ~91% of theoretical capacity, cf. Table S1) and excellent cycling over 300 cycles (99.2% at 0.5 C and 97.3% at 20 C). Moon et al. attributed this to the encapsulated monoclinic phase, pore confinement, and thin carbon shells, which jointly suppressed polysulfide dissolution and buffered volume expansion [51]. This study provided the first experimental evidence that monoclinic sulfur can remain stable in a carbon host. Still, the specific mechanistic contribution of the monoclinic phase remained unclear, as being closely linked to the broader effect of geometric confinement, rather than being unique to the monoclinic form itself.

Peng et al. [54] synthesized an ultrathin, graphene-templated microporous carbon, featuring highly uniform 0.5–0.6 nm pores, which were shown to be small enough to block entering cyclo-S_8_ species but at the same time allowing S_2_–S_4_ chain species to enter. Sulfur was incorporated by melt impregnation up to ~50–60 wt.%, and XRD showed no crystalline S_8_ peaks below about 50 wt.% sulfur loading. As in the works of Xin et al. and Li et al., it was also concluded here that sulfur trapped in the pores is primarily present as the smaller S_2_–S_4_ molecules, with any remaining S_8_ located outside the micropores. The constructed Li-S cell exhibited a single discharge plateau at ~1.6–1.8 V and a single charge plateau at ~2.0–2.2 V, with an initial capacity of ~1350 mAh g^−1^, consistent with a direct S_2_–S_4_ → Li_2_S conversion rather than the multi-step polysulfide cascade. The cathode combined high rate capability (~710 mAh g^−1^) with good stability (~900 mAh g^−1^ after 150 cycles), which was linked to the successful confinement of small sulfur species that suppressed the formation of soluble polysulfides. Similarly to the previous works of Xin et al. [52] and Li et al. [53], this study considered sulfur from the point of view of its molecular size and did not address differences in α-β-γ sulfur allotropy.

Jung and Han [55] offered a theoretical insight into the formation of monoclinic sulfur, clarifying the stabilizing role of carbon. Using DFT and ab initio molecular dynamics, they showed that in the absence of carbon, orthorhombic sulfur is more stable at ambient conditions, and lithiation alone does not drive an orthorhombic → monoclinic transition. However, when ~0.3 carbon atoms per S8 unit were incorporated into the sulfur cathode, an orthorhombic → monoclinic transition of sulfur occurs, suggesting that carbon acts not merely as a conductive scaffold but as a structural co-component of the forming sulfur lattice via formation of stable –S–C–C–C–S– chains. This theoretical study highlighted the control of crystal phases through composite chemistry as a promising strategy for designing Li–S cathodes, alongside pore-size and S_2_–S_4_ confinement methods.

Fang et al. [56] addressed the key practical limitation of Li–S batteries, i.e., low sulfur content and areal loading, fabricating electrodes with sulfur contents exceeding 90 wt.% using SWCNT as conductive and nanoporous host providing dense coatings and sulfur crystallites intimately contacted with the nanotubes. Collected XRD patterns revealed that the cathode contains mainly monoclinic γ-sulfur, and α-sulfur is formed only at very high loadings as excess, poorly contacted sulfur agglomerates due to γ-S → α-S reconversion. Electrochemically, the 95 wt.% S electrode (2.4 mg S cm^−2^) delivered ~1280 mAh g^−1^, retaining ~70% of this capacity after 300 cycles. In contrast to previous works, two discharge plateaus were observed at all tested current densities and remained well defined even at high rates and high areal sulfur loadings. Thus, the proposed electrode architecture clearly promoted the formation and stabilization of γ-sulfur, but it did not suppress the classic two-plateau S_8_ → Li_2_S_n_ → Li_2_S behavior, indicating that soluble polysulfides could still play a significant role in the reaction mechanism.

Helen et al. [57] revisited the long-standing assumption that sulfur confined in ultramicroporous carbon exists as small S_2_–S_4_ species undergoing a polysulfide-free mechanism. They synthesized ultramicroporous carbon with ~0.53 nm pores, which was infiltrated with sulfur by melt infusion at 155 °C under vacuum. Thanks to combined XPS, EELS, in situ Raman, and in situ EIS studies, it was possible to determine the actual sulfur speciation and the Li–S reaction pathway in ether- and carbonate-based electrolytes. Contrary to earlier interpretations based mainly on nitrogen sorption and ex situ XPS, it was shown here that sulfur inside the ultramicropores did not form discrete S_2_–S_4_ species, but rather existed as linear polymeric sulfur chains. Lithium reacting with confined sulfur followed a single voltage plateau (~1.8/~2.2 V on discharge/charge), which was not attributed to a change in sulfur molecular allotropy, but to effective size exclusion of solvent molecules. Ultramicropores physically separated sulfur from the liquid electrolyte, suppressing the dissolution of polysulfides and enabling operation in carbonate electrolytes without classical shuttling. When the same composite was cycled in ether LiNO_3_-free electrolyte, three discharge plateaus in the first cycles were observed: two at ~2.4–2.3 V and 2.0–2.1 V corresponding to formation of higher- and lower-order polysulfides (i.e., conventional S_8_ chemistry in regions accessible to solvent), and a third, lower-voltage plateau below 2.0 V, assigned to a solid–solid conversion of sulfur confined deep in the ultramicropores. Overall, Helen et al. doubted the previous confinement pictures introduced, arguing that the key factor was polymeric sulfur immobilized by strict pore-size exclusion of solvent. The size of the carbon pores was mentioned to play a critical role in determining the reaction path of lithium with sulfur confined within these pores. Nevertheless, despite the strong materials characterization, the electrochemical investigation is limited to basic tests, providing insufficient mechanistic insight into the role of sulfur speciation.

Li et al. [58] approached the problem of shuttle suppression by sulfur incorporation into a high-sulfur organosulfur polymer composite, in which ultrafine monoclinic S nanocrystals are embedded within a polymer matrix. This architecture enables the achievement of a very high sulfur content (90 wt.%, cf. Table S1, Supplementary Materials), yet without any porous carbon able to confine short S_x_ chains. Electrochemically, such a cathode exhibited a quasi-single-plateau behavior showing a dominant discharge plateau in the typical 1.8–2.0 V region and stable capacities of ~800 mAh g^−1^ at 0.5 C over 200 cycles, suggesting that polysulfide loss is strongly suppressed despite the absence of a strictly confined sulfur species. The authors explicitly attribute the improved electrochemical performance to the presence of monoclinic sulfur and its intimate integration with the organosulfur framework, emphasizing crystal phase and chemical bonding as the key design levers rather than sub-nanometer pore size. Unfortunately, the authors did not perform electrochemical tests in carbonate-based electrolytes, which prevents a direct comparison of their results with other discussed studies, where such electrolytes were used (e.g., [52]). Li et al. complement their electrochemical results with in situ UV spectroscopy, showing that monoclinic sulfur domains and the organosulfur framework remain structurally coherent over cycling, and that the main redox process proceeds without a dominant contribution from soluble long-chain polysulfides. Consequently, this work provides an important counterpoint to the pore-size-driven nanoconfinement mechanisms and demonstrates that high sulfur loading, single-plateau-like behavior and good cycling stability in Li-S batteries can be achieved in the absence of a dedicated ultramicroporous carbon host [58].

Grabe et al. [59] combined modeling and experiment to understand how molten sulfur infiltrated porous carbon hosts and which sulfur allotropes formed inside different pore sizes during this process; a highly microporous carbon textile with pore-size peaks at ~0.7 and ~1.3 nm, and powdered activated carbon with a bimodal pore distribution (micropores at ~0.6 nm and ~0.9 nm with additional ~50 nm mesopores. The simulations predicted that in ultramicropores the formation of linear S_4_ and S_6_ chains was thermodynamically and energetically favored. Cyclic S_8_ species were repelled from the smallest pores, while they could reside without scission in larger micro/mesopores. The collected XPS and Raman spectra supported the model predictions: linear S_4_ and S_6_ species were detected and attributed to sulfur in ultramicropores. In contrast, conventional S_8_ species dominated in larger pores and on external surfaces. This work did not study electrochemical performance but rather provided a mechanistic justification for the formation of short sulfur chains in ultramicroporous hosts and led to the mitigation of polysulfide shuttling.

Pai et al. [60] used a freestanding binder and current-collector-free carbon nanofibers (as the host and deposited sulfur from the vapor phase, followed by slow cooling. Structural and textural characterization showed that about 50 wt.% sulfur was loaded; however, a substantial fraction of sulfur remained “pore-unconfined” rather than being loaded on the external surface. Surprisingly, XRD revealed that this surface-located sulfur adopted was room temperature stable γ-S_8_ phase, stable at room temperature over at least one year, with no detectable reversion to the orthorhombic α-S_8_ phase. The electrochemical cells based on γ-S/CNF cathodes and carbonate-based electrolytes displayed a single discharge plateau and an altered redox mechanism. After an initial activation, the capacity stabilized at around 800 mAh g^−1^, followed by exceptionally stable cycling over 4000 cycles, finally still retaining about 650 mAh g^−1^. Careful electrochemical and post-mortem spectroscopic/microscopic analyses suggested a direct and reversible conversion of monoclinic γ-S to Li_2_S without detectable formation of soluble polysulfide intermediates, despite the predominant unconfined sulfur. In contrast to earlier strategies that demanded sub-nanometer pores and strict molecular-size confinement [52,53,54,57], this study demonstrated that the crystal phase of sulfur itself, i.e., γ-sulfur stabilized on a suitable carbon nano-scaffold—can provide a polysulfide-free, single-plateau behavior in carbonate electrolytes, opening a conceptually new route towards practical, high-energy Li-S batteries based on carbonate electrolytes.

Pai et al. [61] extended their findings from Li-S to K-S batteries, demonstrating that a non-confined γ-monoclinic sulfur phase can also enable long-life K-S cells in commercially favorable carbonate electrolytes. The same cathode design yields ~50 wt.% sulfur loading and ~1 mg S cm^−2^ areal loading and was paired with a potassium metal anode and carbonate-based electrolyte that normally fails rapidly in K-S due to polysulfide–carbonate side reactions. Cyclic voltammetry showed an initial irreversible reduction peak at ~0.3 V, followed by a broad reduction around 0.5 V and a broad oxidation at ~2.3 V, consistent with an overlapped sequence of K-polysulfide conversions rather than well-resolved plateaus. Under galvanostatic cycling, the cell delivered ~900–925 mAh g^−1^, retaining ~95% of this capacity over 500 cycles with a single, smoothly sloping discharge profile. Post mortem XPS confirmed that γ-sulfur was reversibly converted via higher-order K_2_S_6_/K_2_S_4_ to K_2_S and reconverted to sulfur, with no strong signatures of dissolved polysulfides and no classical upper-voltage plateau, implying a predominantly solid-state γ-S → K-polysulfides/K_2_S pathway, which permits the use of carbonate electrolytes without severe shuttle. This important work showed that a non-confined γ-sulfur phase may be used for polysulfide mitigation in other metal–sulfur systems beyond lithium.

Barczak et al. [62] introduced sulfur-tuned advanced carbons (STACs) prepared via a new steam-assisted sulfur insertion (SASI) method that aimed to combine very high sulfur loadings with precise, pore-size-dependent sulfur location. By tuning temperature, pressure, and exposure time, the authors could confine sulfur into specific pore ranges and adjust which fraction of micro-, meso-, and macroporosity remained open. The nominal sulfur loading was parametrized as a fraction of the initial pore volume of carbon host (Black Pearls 2000, V_T_ = 2.5 cm g^−1^), and spanned seven S-doping levels: 0, 2.5, 5, 10, 25, 50, and 100% of the initial V_T_. Thermogravimetric analysis and elemental analysis showed that the actual sulfur mass fraction increased from ~11 wt.% (2.5% doping level) to ~85 wt.% (100% doping level), with almost complete loss of S_BET_ and V_T_ for the latter. Four-point-probe measurements revealed that the electrical conductivity of STAC-0 (pure carbon) decreased significantly only when sulfur loadings exceeded 70 wt.%, indicating that sulfur pore insertion does not completely interrupt electron pathways. Structural characterization confirmed that a significant fraction of the confined sulfur crystallized was present as monoclinic γ-sulfur. Barczak et al. showed their possible use as cathodes in rechargeable Al-S, though the paper focused on synthesis, structure, and multifunctional applications rather than dissecting in detail Al-S electrochemistry or the role of γ-S in suppressing polysulfides.

Building on this, Pauletto et al. [63] provided a mechanistic view of how such STACs stabilize γ-sulfur as a function of pore architecture, surface chemistry, and defect structure. Using the same SASI approach applied to other carbon matrices, it was shown that sulfur was distributed hierarchically from ultramicropores to mesopores and crystallized as mixtures of α-S, γ-S, and, in the smallest pores, short S_x_ fragments, with the γ-S fraction increased in hosts that were more basic, more conductive, and richer in defects. The collected results indicated that beyond pore-size control, electronically conductive, defect-rich carbon environments may actively promote γ-S nucleation and kinetic stabilization, helping to rationalize the design of future γ-S-based sulfur cathodes.

Recently, Nishanth et al. [64] developed a chemical-activation route to load large amounts of monoclinic γ-sulfur onto electrospun PAN-derived carbon fiber mats for Li–S cathodes operating in carbonate electrolytes. The proposed fabrication protocol yielded non-confined γ-S coatings on both pristine and activated supports; in the case of the latter, nearly a four-fold increase in monoclinic sulfur was observed. Li-S coin cells assembled with γ-S-loaded ACF cathodes, lithium metal anodes, and a low-cost carbonate electrolyte delivered a specific capacity of ~970 mAh g^−1^ (areal capacity ≈ 1 mAh cm^−2^) and retained about 91% of the initial capacity after 1000 charge–discharge cycles. Interestingly, at very high rates (up to 40 C), the cells still had a specific capacity of ~400 mAh g^−1^, demonstrating exceptional rate capability compared with most carbonate-based Li-S systems. Electrochemical analyses of cycled cells, complemented by post-mortem characterization, indicated a modified redox mechanism in which γ-sulfur is reversibly and directly converted to Li_2_S with little or no presence of soluble polysulfide intermediates. This study showed that non-confined gamma sulfur may provide long-life, high-rate operation in practical carbonate electrolytes, bringing γ-S cathodes a step closer to commercial relevance.

Summarizing the discussion provided in this chapter, the observed single-plateau behavior testifying suppression of the shuttle effect is attributed to two partially overlapping, but mechanistically distinct concepts. Some studies explain is that size exclusion and sub-nanometer confinement stabilizing short-chain sulfur species (S_2_–S_4_) and effectively separating the active material from solvent molecules, leading to a quasi-solid-state S_x_ ↔ Li_2_S pathway with strongly hindered polysulfide transport [52,53,54,57].

In contrast, other studies [51,60,61,64] ascribe this single-step conversion in carbonate electrolytes to the presence of a long-range-ordered monoclinic sulfur phase, supported by DFT arguments that carbon penetration into the S_8_ lattice stabilizes the γ-phase and may modify the redox pathway. Importantly, none of these reports provide a fully articulated mechanistic in situ/operando evidence for how the monoclinic crystal structure itself could suppress polysulfide formation, beyond the empirical correlation between γ-phase stabilization with single-plateau voltage profiles and ex situ evidence for Li_2_S presence as the dominant discharge product.

## 5. Methodological Limitations in Sulfur Electrochemistry

Despite the rapidly growing number of studies on the role of γ-sulfur and/or nanoconfined sulfur species, the available experimental evidence remains fragmented and often indirect, due to critical limitations of the available ex situ characterization techniques. In this context, the viable methods used to monitor the state of the sulfur and resulting reaction pathways would be of paramount importance, expanding conventional electrochemical measurements such as cyclic voltammetry, galvanostatic discharge–charge profiles, and impedance spectroscopy, as well as classical ex situ methods (XRD, XPS, and TGA); both provide useful but indirect information about the underlying sulfur speciation.

In most reports on γ-S/nano-S, phase and speciation assignments rely primarily on ex situ powder XRD [51,60,62,63]. However, this technique is intrinsically sensitive only to crystalline domains that extend over many unit cells; therefore, it is effectively blind to non-crystalline short S_x_ fragments/chains confined in ultramicropores lacking three-dimensional periodicity. In carbon hosts, this effect is further amplified by the scattering from the disordered carbon matrix, which masks any weak diffuse signal from nanoconfined sulfur. As a result, the quantitative fraction of γ-S or short S_x_ fragments is at least poorly constrained, most often even not possible. For example, Barczak et al. [62] observed that well-resolved γ-S reflections become visible only at very high sulfur contents (typically > 50 wt.%), despite clear evidence from other techniques that sulfur is present within the nanopores.

Beyond XRD, several techniques are routinely used to probe sulfur in carbon matrices, each offering valuable information but also suffering from important limitations. X-ray photoelectron spectroscopy (XPS), typically probing only the top few nanometers of the surface, is widely used to identify sulfur oxidation states and bonding environments [59]. XPS spectra primarily detect sulfur exposed at or near the external surface and in larger pores, and underestimate the total amount of sulfur. Moreover, XPS—being sensitive to chemical states and local bonding environments (e.g., sulfide, polysulfide, sulfate)—is not able to distinguish between different crystallographic forms of sulfur (α-, β-, γ-sulfur).

Thermogravimetric analysis (TGA) and differential scanning calorimetry (DSC) are very useful for determining total sulfur content and for identifying phase transitions or decomposition events, but cannot credibly distinguish between crystalline, amorphous and nanoconfined populations of sulfur within the carbon matrix [62,63]. Used in combination, XRD, XPS, Raman and TGA/DSC can yield a reasonably coherent qualitative picture of sulfur speciation and overall loading, but they still fall short of providing a complete, spatially resolved and quantitatively robust description of γ-sulfur and nanoconfined sulfur species in porous carbons.

Raman spectroscopy provides complementary vibrational fingerprints that can distinguish different sulfur species, such as cyclo-S_8_ and various polysulfides, and track their evolution during cycling. Characteristic Raman bands have been assigned to S_8_ rings, long-chain polysulfides (e.g., S_8_), and shorter S_2_–S_4_ species, and in situ Raman measurements can be used to follow the appearance and disappearance of these species during electrochemical testing [65]. Similarly to XRD, Raman signals from sulfur confined in carbon hosts are can be often superimposed by carbon bands; interpretation of band intensities is thus complicated due to weak spectral signals and poor spatial resolution [66,67]. Nevertheless, in situ spectroscopic techniques have already shown some potential in analyzing the real redox mechanisms in Li–S systems [67]. Because each in situ optical spectroscopy method has its own limitations, a multimodal strategy combining two or more complementary in situ techniques definitely would offer a more reliable way to track intermediate discharge products and the corresponding electrochemical processes. This underscores the need for more systematic use of in situ/operando methods (e.g., Raman or UV spectroscopies), which could track phase evolution and sulfur speciation under realistic cycling conditions and thereby provide a more robust basis for distinguishing between competing interpretations.

## 6. Perspectives and Conclusions

The stabilization of γ-sulfur in porous carbon hosts has emerged as one of the most conceptually exciting directions in metal–sulfur battery research. It challenges the long-standing view that soluble polysulfides are an unavoidable feature of sulfur electrochemistry and points toward a future in which industrially relevant carbonate electrolytes could be used with sulfur cathodes. If this potential can be fully realized, it would remove two of the main barriers towards the commercialization of metal–sulfur batteries: the polysulfide shuttle and the dependence on expensive, flammable ether electrolytes.

At the same time, current γ-S research is still in its early stages, and several critical questions remain open. On the characterization side, most reports identify γ-sulfur primarily through conventional ex situ techniques (XRD, XPS, TGA) often at relatively low sulfur loadings where peaks are weak and overlap with other phases. Additionally, these techniques do not provide an unambiguous confirmation of the presence of nanoconfined sulfur, as they cannot directly resolve whether the detected sulfur species are confined within sub-nanometer pores or other regions of the carbon matrix. Therefore, a more rigorous, multi-technique toolbox is needed to unambiguously confirm γ-S/nano-S and track its evolution during cycling. This likely requires combining higher-sensitivity diffraction, operando Raman spectroscopy, and advanced solid-state NMR, together with well-defined reference spectra for γ-S in carbon composites.

Mechanistically, it is not yet clear whether the unusual electrochemical behavior observed for γ-S/carbon electrodes arises uniquely from the γ-allotrope or from geometric confinement and interfacial effects that could, in principle, be achieved with other sulfur forms. One key question is whether polysulfide-free (or strongly suppressed) cycling truly requires γ-S, or whether deeply confined short S_2_–S_4_ chains in sub-nanometer pores would offer similar benefits. Carefully designed model systems that could decouple allotropy (α-S vs. γ-S) from pore size, surface chemistry, and defect density would be essential to address this fundamental question.

A further challenge is translating proof-of-concept cells into practically relevant cathodes. Several γ-S systems are showing excellent cycle life and compatibility with carbonate electrolytes, but typically at moderate sulfur areal loadings and controlled laboratory temperatures. For real applications, cathodes must maintain their performance at higher sulfur loadings and elevated temperatures, while still keeping sufficient rate capability and acceptable safety. It is not yet known whether γ-sulfur remains structurally and electrochemically stable under such demanding conditions, or whether it gradually relaxes to other allotropes.

Finally, scalability and process integration must be considered. Existing γ-S synthesis protocols demonstrate clear feasibility but are not yet fully optimized for large-scale manufacturing. More recent approaches (e.g., steam-assisted sulfur insertion into porous carbons) begin to address this gap by offering controllable, high sulfur loadings in a more industrially plausible, solvent-free process. Future work should focus on adapting γ-S formation to industrial electrode fabrication processes, ensuring compatibility with current Li-ion battery production lines.

In summary, γ-sulfur stabilization in carbon matrices introduces a genuinely new design variable for metal–sulfur batteries. Control over the allotropic form of sulfur, along with proper nanoconfinement, can tune electrochemical reactivity and has the potential to reframe cathode design for metal–sulfur batteries. Whether γ-sulfur will ultimately emerge as a general design principle for metal–sulfur batteries, or rather remain a solution restricted to specific carbon architectures and operating windows, will depend on three advances: (i) unambiguous mechanistic clarification, including the relative roles of γ-S versus deeply confined S_2_–S_4_ chains, (ii) widely adopted and quantitative characterization standards including in situ/operando studies, and (iii) demonstration of high-loading, industrially compatible γ-S cathodes that operate reliably under realistic conditions including temperature, current density, and electrolyte composition.

## Figures and Tables

**Table 1:** Projected global Li-ion battery demand by region and sector (data taken from [1]); reported values are in GWh.

**Demand by Region**	**2022**	**2025**	**2023**
China	400	750	1900
Europe	150	400	1300
United States	100	300	900
Rest of the world	50	250	600
**Total**	**700**	**1700**	**4700**
**Demand by Sector**	**2022**	**2025**	**2030**
Mobility	600	1500	4300
Stationary storage	50	150	300
Consumer electronics	50	50	100
**Total**	**700**	**1700**	**4700**

**Table 2:** Comparison of Li-ion, Li-S and Al-S batteries [12,21,22,23,24,25,26,27,28,29].

	Li-ion	Li-S	Al-S
Theoretical specific capacity (cathode) *	372 mAh g^−1^	~1675 mAh g^−1^	~1675 mAh g^−1^
Theoretical cell energy density ^$^	~900 Wh kg^−1^	~2600 Wh kg^−1^	~1400 Wh kg^−1^
Reported cell energy density ^#^	~250–300 Wh kg^−1^	~400–500 Wh kg^−1^	~90 Wh kg^−1^
Main advantage(s)	High efficiency, Long cycle life	High energy density, lower environmental footprint	Medium energy density, lower environmental footprint
Main disadvantage(s)	Low energy density	Polysulfide shuttle resulting in limited cyclability	Polysulfide shuttle resulting in limited cyclability
Technological maturity	Fully commercial	Pilot-scale prototypes	Early research stage

* Values correspond to the theoretical capacities of the limiting active electrode materials: graphite for Li-ion batteries and sulfur for Li-S and Al-S batteries. ^$^ Theoretical cell energy densities are idealized values and should not be compared directly with practical or demonstrated cell-level metrics. ^#^ Practical cell energy densities correspond to experimentally demonstrated or commercially realized systems. For Al-S, currently demonstrated pouch-cell energy density is ~90 Wh kg^−1^, highlighting the large gap between theoretical benchmark and actual values.

## Data Availability

No new data were created or analyzed in this study. Data sharing is not applicable to this article.

## Supplementary Materials

The following supporting information can be downloaded at: https://www.mdpi.com/article/10.3390/ma19163537/s1, Table S1. Comparison of reported cathode compositions, electrochemical testing conditions and performance metrics for the discussed works.

## Abbreviations

The following abbreviations are used in this manuscript:

Li-S	Lithium–sulfur batteries
Li-ion	Lithium-ion batteries
Mg-S	Magnesium–sulfur batteries
K-S	Potassium–sulfur batteries
Al-S	Aluminum–sulfur batteries
α-S	Alpha sulfur (α-sulfur)
β-S	Beta sulfur (β-sulfur)
γ-S	Gamma sulfur (γ-sulfur)
nano-S	Nanoconfined sulfur
NMC	Nickel–manganese–cobalt
HRTEM	High-resolution transmission electron microscopy
FDUs	FDU-type ordered mesoporous carbons
XRD	X-ray diffractometry
XPS	X-ray photoelectron spectroscopy
TGA	Thermogravimetric analysis
DTG	Differential scanning calorimetry
DFT	Density functional theory
SWCNTs	Single-walled carbon nanotubes
STACs	Sulfur-tuned advanced carbons
SASI	Steam-assisted sulfur insertion
