# Supplementary material for: The Forgotten Allotrope: γ-Sulfur Stabilization in Carbon Matrices for Energy Storage Applications
Source: Materials (Basel). 2026 Aug 20;19(16):3537. doi: 10.3390/ma19163537 (PMC13514742; doi:10.3390/ma19163537)
Supplement: Supplementary file 1 [file materials-19-03537-s001.zip › materials-4440719-supplementary.pdf]

The Forgotten Allotrope: γ-Sulfur Stabilization in Carbon Matrices for Energy Storage Applications

Marlena Bytniewska[1], Dimitrios Giannakoudakis[1] and Mariusz Barczak[1,*]

Institute of Chemical Sciences, Faculty of Chemistry, Maria Curie-Sklodowska University, Lublin, Po-land

*Correspondence: mariusz.barczak@mail.umcs.pl

SUPPLEMENTARY INFORMATION

Table S1. Comparison of reported cathode compositions, electrochemical testing conditions and performance metrics for the discussed works

**Reference**	**Carbon host**	**Sulfur form**	**Achieved sulfur loading [wt.%]**	**Electrolyte (E) and E/S ratio**	**Tempe rature**	**Current density / C-rate** and rate capabilities [mAh g^{-1}]	**Capacity retention**	**Key observations**
**Moon et. al. [51]**	Carbon nanotubes' shell formed inside anodic aluminum oxide template<br><br>Porous characteristic not provided	Mainly monoclinic sulfur near CNT walls stabilized by the carbon-lined AAO nanochannels and orthorhombic sulfur in the core	~80	1M LiTFSI in DOL/DME 1:1 (v/v)<br>E/S ratio not provided	25 °C	**0.5-40 C**<br>**1520** @ 0.5 C<br>**1234** @ 20 C<br>**453** @ 40 C	After 300 cycles:<br>**99.2 %** @ 0.5 C<br>**97.3 %** @ 20 C	Encapsulated monoclinic γ-S near CNT walls and orthorhombic S in the core are observed inside AAO nanochannels, without strict sub-nanometer nanoconfinement. Single-plateau behavior in some conditions and excellent rate capability (up to 40 C) are attributed to altered sulfur phase and improved transport, but the authors do not provide a detailed mechanistic model for γ-S stabilization or explicitly separate polysulfide-free solid-state conversion from a conventional Li_2S_n shuttle.<br>*Characterization techniques:* XRD and HRTEM
**Xin et al. [52]**	Core/shell carbon host:<br>Core: multiwalled carbon nanotubes<br>Shell: microporous carbon (pore size ~0.5 nm)	Confined small sulfur allotropes $S_{2\text{-}4}$ without allotrope speciation	~40	0.5 M LiTFSI in tetra(ethylene glycol) dimethyl ether<br>- and -<br>1 M $LiPF_6$ in EC/DMC 1:1 (v/v) (CE-carbonate electrolyte)	n/p	**0.1-5 C**<br>**1670** @ 0.5 C in carbonate electrolyte<br>**800** @ 5 C in carbonate electrolyte	After 200 cycles:<br>**100 %** @ 0.1 C	Very well evidenced sulfur nanoconfinement without allotrope speciation. Well supported evidence that short S_2–S_4 sulfur species significantly suppress polysulfide formation. High specific capacity of 1670 mAh g^{-1}, very good cycling stability and a favorable high-rate capability of 800 mAh g^{-1} at 5 C

	S_{BET}=936 m^2 g^{-1}, d_{DFT} =0.5 nm			E/S ratio not provided				*Characterization techniques:* HRTEM, SEM, Raman, N_2 sorption, XRD, XPS
**Li et al. [53]**	Ordered microporous carbon FDU, pore size ~0.46 nm	Confined small sulfur allotropes S_{2-4} without allotrope speciation	~60	1 M $LiPF_6$ in EC/DMC (1:1 v/v)<br>- and -<br>1 M LiTFSI in DOL/DME (1:1 v/v) + 0.2 M $LiNO_3$<br>E/S ratio not provided	RT	**0.1-3 Ag^{-1}**<br>**~1150** @ 0.1 Ag^{-1}<br>**~750** @ 1 Ag^{-1}<br>**~400** @ 3 Ag^{-1}	After 400 cycles:<br>**~ 78 %** @ 1 Ag^{-1}	Very well evidenced sulfur nanoconfinement without allotrope speciation.<br>Well supported evidence that short S_2–S_4 sulfur species significantly suppress polysulfide formation<br>*Characterization techniques:* HRTEM, SEM, XRD, XPS , TGA, N_2 sorption
**Peng et al. [54]**	Graphene-based ultrathin microporous carbon with pore size 0.58–0.68 nm; thickness ~30–60 nm, synthesized by glucose hydrothermal carbonization and self-assembly	Smaller sulfur molecules S_2–S_4 confined within the ~0.6 nm micropores	~50	1 M LiTFSI in DOL/DME (1:1 v/v) + 0.1 M $LiNO_3$<br>E/S ratio not provided	20 °C	**0.1-3 C**<br>**~1350** @ 0.1 C<br>**~850** @ 1 C<br>**710** @ 3 C	After 150 cycles:<br>~ **67 %** @ 0.1 C	Evidenced sulfur nanoconfinement without allotrope speciation.<br>Well supported evidence that short S_2–S_4 sulfur species significantly suppress polysulfide formation<br>*Characterization techniques:* HRTEM, SEM, XRD, XPS , TGA, N_2 sorption
**Fang et al. [56]**	Freestanding single-wall carbon nanotube network film with very low areal density (~0.12 mg cm^{-2}), acting as an ultralight, highly conductive host for sulfur impregnation and forming an almost pure sulfur electrode.	Monoclinic γ-S (γ-S_8) stabilized in carbon nanotube network	~95	1 M LiTFSI + 0.2 M $LiNO_3$ in DOL/DME (1:1 v/v)<br>E/S ratio not provided	RT	**0.25-1.68 Ag^{-1}**<br>**~1280** @ 0.25 A g^{-1}<br>**~900** @ 1.68 A g^{-1}	After 100 cycles:<br>**84.5%** @ 2.4 mg_S cm^{-2}<br>*(2.4 mg_S cm^{-2} corresponds to ~1200 mAh g^{-1} based on sulfur)*<br>**77.7%** @ 4.8 mg_S cm^{-2}<br>**70.0%** @ 7.2 mg_S cm^{-2}	A highly conductive, ultralight SWCNT network enables both very high sulfur content (95 wt.%) and high areal sulfur loading (7.2 mg cm^{-2}) while maintaining high areal capacity (8.63 mAh cm^{-2}), illustrating that electronic conduction efficiency of the carbon host is a critical parameter for designing practical high-energy Li–S cathodes with minimal inactive mass.<br>*Characterization techniques:* SEM, TEM, XRD, Raman, TGA, N_2 sorption, four-point probe
**Helen et. al [57]**	Ultramicroporous carbon (UMC) obtained by KOH activation of coconut-shell-derived carbon (S_{BET} ≈ 1600	Linear polymeric sulfur chains confined in ultramicropores; with no	~45	1.0 M $LiPF_6$ in EC/DMC (1:1 v/v) (carbonate electrolyte)<br>- and –	RT	**No rate capability study, only C/20 was applied**<br>**~1200** @ 0.05 C	After 60 cycles:<br>~ **30 %** @ 0.05 C	Sulfur confined in ultramicroporous carbon (0.5–0.7 nm) is proposed to exist as linear polymeric chains rather than shorter S_2–S_4 fragments. The abundant ultramicropores limit solvent access, suppress polysulfide dissolution and enforce a quasi-solid-state conversion mechanism with a

	$m^2\ g^{-1}$, $V_T \approx 0.66\ cm^3\ g^{-1}$, main pore size ≈ 0.5–0.7 nm).	spectroscopic evidence for smaller S_2–S_4 chains.		1.0 M LiTFSI in DOL/DME (1:1 v/v) (ether electrolyte); E/S ratio not reported.				single voltage plateau in carbonate electrolyte, but the authors do not attempt to identify γ-S and do not propose a specific sulfur allotrope; they focus on pore-confinement-driven suppression of Li_2S_n shuttle. *Characterization techniques:* HRTEM, SEM, XRD, XPS , TGA, N_2 sorption
**Li et al. [58]**	Organosulfur polymer composite with sulfur chemically and physically integrated within the triallyl isocyanurate-based polymer matrix. Conductive carbon is only present as a conventional conductive additive in the electrode formulation, and not as nanoconfining carbon framework.	Monoclinic non-confined sulfur nanocrystals embedded in an organosulfur polymer matrix.	90	1 M LiTFSI + 2 wt.% $LiNO_3$ in DOL/DME (1:1 v/v) E/S ratio: 25	RT	**0.5–3 C** **1126** @ 0.2 C **958** @ 0.5 C **789** @ 1 C **747** @ 3 C	**91 %** after 200 cycles @ 0.5 C; **94 %** after 350 cycles @ 1 C.	Embedding monoclinic sulfur nanocrystals within an organosulfur polymer matrix enables simultaneously very high sulfur loading (90 wt.%), suppression of polysulfide dissolution, and improved cycling stability. They do not test carbonate electrolytes; only ether-based electrolyte. *Characterization techniques:* FTIR, TGA, HRTEM, XRD, **in-situ UV.**
**Pai et. al [60]**	Activated freestanding carbon nanofibers binder-free and current-collector-free	monoclinic non-confined γ-S_8 sulfur (stabilized at RT on CNFs)	~50	1 M LiPF6 in EC:DEC (1:1 v/v) - and - EE in 1 M LiTFSI in DME:DOL (1:1) + 1 wt.% LiNO3 E/S ratio: 20	RT	**0.5–40 C** **1170** @ 1 C **980** @ 5 C **900** @ 10 C **410** @ 40 C	After 4000 cycles: ~ **82 %** @ 0.5 C (i.e. ~0.04% decay per cycle)	Free-standing γ-S/CNF cathodes (non-confined monoclinic γ-S stabilized at RT on CNFs) enable long-term cycling in carbonate electrolyte, delivering ~800 mAh g^{-1} in early cycles and ~658 mAh g^{-1} after 4000 cycles at 0.5 C (~0.04% decay per cycle). The authors attribute the single or gently sloping discharge profile and exceptional cyclability to a γ-S-mediated reaction pathway in carbonate electrolyte, but conclude that the cells still follow a polysulfide digression route rather than a purely polysulfide-free solid-state mechanism. γ-S is non-confined on CNFs, and stabilization is attributed to carbon environment and synthesis conditions rather than sub-nanometer pores. *Characterization techniques:* XRD, SEM, TEM, XPS, TGA, N_2 sorption

**Pai et. al [61]*** *K-S batteries were studied here, not Li-S batteries	Activated freestanding carbon nanofibers binder-free and current-collector-free	monoclinic γ-sulfur, γ-S_8 (stabilized at RT on CNFs)	~50	1 M KPF_6 in EC:DEC (1:1 v/v) E/S ratio: 20	RT ~25 °C	**0.05–2 C** **~950** @ 0.05 C **~800** @ 0.1 C **~250** @ 1 C **~90** @ 2 C	After 500 cycles: **95 %** @ 0.1 C	Non-confined monoclinic γ-S_8 stabilized on activated CNFs enables stable room-temperature K–S cells with a commercial carbonate electrolyte, delivering ~950 mAh g^{-1} at 0.1 C and retaining ~95% of this capacity after 500 cycles. The authors propose that γ-S, even in a non-confined state, facilitates a largely direct solid-state S ↔ K_2S conversion with a single sloping discharge profile and greatly reduced soluble K-polysulfide formation, demonstrating that carbonate electrolytes can be used in K-S systems without strict sulfur nanoconfinement. *Characterization techniques:* XRD, SEM, TEM, XPS
**Nishanth et. al [64]**	KOH-activated PAN-derived carbon fibers obtained from electrospun PAN mats (S_{BET}=162 m^2 g^{-1} and micropore fraction: 45.6%)	Monoclinic γ-S_8 (space group P2/c) deposited via vapor-phase deposition forming a non-confined γ-sulfur coating	~25	1 M $LiPF_6$ in EC:DMC (1:1 v/v) E/S ratio: 25	RT ~25°C	**1–40 C** **900** @ 1 C **400** @ 40 C	After 1000 cycles: **91 %** @ 1C	KOH activation of PAN-derived carbon fibers increases monoclinic γ-sulfur loading nearly fourfold by boosting surface area and microporosity, while preserving a non-confined γ-S architecture that still supports a modified redox mechanism consistent with reversible γ-S to/from Li_2S conversion and minimal formation of soluble polysulfides in carbonate electrolytes. *Characterization techniques:* SEM, TEM, XRD, XPS, TGA, N_2 sorption
